# Human MHC Class II and Invariant Chain Knock‐in Mice Mimic Rheumatoid Arthritis with Allele Restriction in Immune Response and Arthritis Association

**DOI:** 10.1002/advs.202401513

**Published:** 2024-04-11

**Authors:** Laura Romero‐Castillo, Taotao Li, Nhu‐Nguyen Do, Outi Sareila, Bingze Xu, Viktoria Hennings, Zhongwei Xu, Carolin Svensson, Ana Oliveira‐Coelho, Zeynep Sener, Vilma Urbonaviciute, Olov Ekwall, Harald Burkhardt, Rikard Holmdahl

**Affiliations:** ^1^ Medical Inflammation Research Division of Immunology Department of Medical Biochemistry and Biophysics Karolinska Institute Stockholm 17177 Sweden; ^2^ Fraunhofer Institute for Translational Medicine and Pharmacology ITMP Fraunhofer Cluster of Excellence for Immune‐Mediated Diseases CIMD Theodor‐Stern‐Kai 7 60596 Frankfurt am Main Germany; ^3^ Department of Pediatrics Institute of Clinical Sciences and Department of Rheumatology and Inflammation Research Institute of Medicine The Sahlgrenska Academy University of Gothenburg Gothenburg 41345 Sweden; ^4^ Division of Rheumatology University Hospital Frankfurt Goethe University 60590 Frankfurt am Main Germany; ^5^ Medical Inflammation Research MediCity Research Laboratory University of Turku Turku FI‐20520 Finland

**Keywords:** autoimmune diseases, CIA, DTH, humanized mice, MHC class II, rheumatoid arthritis

## Abstract

Transgenic mice expressing human major histocompatibility complex class II (MHCII) risk alleles are widely used in autoimmune disease research, but limitations arise due to non‐physiologic expression. To address this, physiologically relevant mouse models are established via knock‐in technology to explore the role of MHCII in diseases like rheumatoid arthritis. The gene sequences encoding the ectodomains are replaced with the human DRB1*04:01 and 04:02 alleles, DRA, and CD74 (invariant chain) in C57BL/6N mice. The collagen type II (*Col2a1*) gene is modified to mimic human COL2. Importantly, DRB1*04:01 knock‐in mice display physiologic expression of human MHCII also on thymic epithelial cells, in contrast to DRB1*04:01 transgenic mice. Humanization of the invariant chain enhances MHCII expression on thymic epithelial cells, increases mature B cell numbers in spleen, and improves antigen presentation. To validate its functionality, the collagen‐induced arthritis (CIA) model is used, where DRB1*04:01 expression led to a higher susceptibility to arthritis, as compared with mice expressing DRB1*04:02. In addition, the humanized T cell epitope on COL2 allows autoreactive T cell‐mediated arthritis development. In conclusion, the humanized knock‐in mouse faithfully expresses MHCII, confirming the DRB1*04:01 alleles role in rheumatoid arthritis and being also useful for studying MHCII‐associated diseases.

## Graphical abstract

Physiologically accurate mouse strains, surpassing the limitations of transgenic approaches, are made with knock‐in technology replacing extracellular domains of HLA‐DRA, HLA‐DRB1*04:01/*04:02, and CD74 in C57BL/6N mice. Distinct roles of DRB1*04:01 and 04:02 alleles in MHCII diseases such as rheumatoid arthritis, are confirmed using a collagen‐induced arthritis model. The strains offer an opportunity to investigate human MHCII‐related diseases in the mouse.

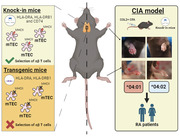

## 1 Introduction

Major chronic immune‐mediated inflammatory disorders, including rheumatoid arthritis (RA), multiple sclerosis (MS), and insulin‐dependent diabetes mellitus, are autoimmune diseases whose susceptibility is intricately linked to specific major histocompatibility complex class II region (MHCII) haplotypes. In humans, the MHCII region is known as the Human Leukocyte Antigen (HLA) class 2 region.^[^ ^1^ ^]^ The pathogenesis of MHCII‐associated autoimmune diseases is dependent on a complex interplay of various genetic and environmental factors. RA, affecting ≈0.5% of the global population, is characterized by inflammation of peripheral joints and consequential bone destruction, driven by synovial tissue infiltration with immune cells, secretion of proinflammatory cytokines, and matrix metalloproteinases.^[^ ^2^ ^]^ While genome‐wide association studies have successfully identified over 100 genetic loci linked to RA, the position and role of the underlying causative polymorphism remain to be fully elucidated. Notably, the strongest genetic association emerges from the MHCII region on chromosome 6, accounting for 30–50% of the total genetic risk of RA.^[^ ^1^, ^3^, ^4^ ^]^ Within the MHCII region, haplotypes containing HLA‐DRB1 alleles are associated with RA, including *04:01, *04:04, *04:05, *04:08, *01:01, *01:02, *14:02, and *10:01 alleles. The DRB1*04:01 association particularly stands out as the predominant risk factor among Caucasians, being detectable in 50% of RA patients. In contrast, the DRB1*04:02 allele belongs to a subset of disease‐protective alleles, such as *04:02, *01:03, *11:02, *11:03, *13:01, *13:02, and *13:23. Consequently, the impact of DRB1 alleles on RA is noteworthy, with *04:01 being the most prevalent among Caucasian RA patients, collectively accounting for 9.7% of the entire genetic variance of RA. This significance is underscored when juxtaposed with the 4% contribution from all non‐HLA risk loci, emphasizing the pivotal role of DRB1 alleles in shaping adaptive autoimmune responses.^[^ ^4^, ^5^ ^]^

Historically, the strong association between different HLA‐DR alleles and positive RA was attributed to the presence of consensus amino acid sequences (QRRAA, RRRAA, and QKRAA) spanning positions 70–74 in the β1 subunit of the HLA‐DR molecule. This phenomenon, encoded by classical haplotypes within the HLA‐DRB1 gene, established the “shared epitope” alleles hypothesis.^[^ ^6^ ^]^ However, this hypothesis does not fully explain the association between HLA‐DRB1 and RA. Notably, newer findings propose that positions 11, 13, 71, and 74 within the DRB1 chain may be intricately linked to RA susceptibility.^[^ ^4^, ^7^ ^]^ Intriguingly, a comparative analysis of these positions between the susceptible *04:01 allele and the protective *04:02 allele unveiled only one distinction: lysine (K) at position 71 for *04:01, as opposed to glutamic acid (E) for *04:02.^[^ ^8^ ^]^ Thus, this amino acid allelic difference could be a starting point to experimentally investigate the intricate mechanisms causing a complex autoimmune disease. Type II collagen (COL2) is expressed not only in cartilage but also in thymus and bone marrow, being a template for MHCII‐associated selection of the immune system.^[^ ^9^, ^10^ ^]^ COL2 also emerges as a self‐antigen involved in arthritis pathogenesis, based on its well‐established role as an arthritogenic immunogen in experimental arthritis.^[^ ^11^, ^12^ ^]^ Furthermore, patients diagnosed with RA exhibit detectable antibodies targeting both native and citrullinated forms of COL2,^[^ ^13^ ^]^ alongside the presence of COL2‐specific T cells.^[^ ^14^ ^]^ Immunization with COL2 leads to the development of collagen‐induced arthritis (CIA) in mice expressing the MHCII allele A^q^.^[^ ^15^ ^]^ Later, it could be shown that transgenic mice expressing DRB1*04:01 were similarly susceptible to CIA. In fact, A^q^ and DR*0401 bind the same immunodominant peptide, COL2_259‐273_.^[^ ^16^, ^17^ ^]^ It is important to note that the major COL2_259‐273_ peptide can undergo hydroxylation and galactosylation at the lysine residue at position 264, which is the key sidechain interacting with the TCR. T cell responses to the galactosylated variant of the 259–273 peptide is predominantly seen in the mouse A^q^ in CIA model as well as in RA, whereas a very strong T cell response to the non‐modified COL2_259‐273_ peptide is predominantly seen in transgenic mouse models expressing DRB1*04:01.^[^ ^18^, ^19^ ^]^ In addition, mouse and human COL2 differ in the immunodominant peptide at position 266, where the mouse has an aspartic acid (D) and the human glutamic acid (E).^[^ ^20^ ^]^ This difference affects the binding to the MHCII molecule, in which the 266E peptide is a better binder than the D266 peptide to the arthritis‐associated MHCII allele A^q^.^[^ ^21^ ^]^ Mice with a mutated COL2, leading to the amino acid replacement of 266E, develop stronger immune tolerance to COL2 and are less susceptible to induction of CIA.^[^ ^22^, ^23^ ^]^ Therefore, humanized COL2^266E^ mice are needed to address the role of human MHCII in mouse models using the CIA model.

Meanwhile, animal models are critical for understanding disease pathogenesis and useful in preclinical testing, the common use of mice with transgenic expression of MHCII is problematic. One notable concern is that the integration of the transgene in the genome leads to aberrant expression and potentially disturbing the normal functioning of other genes. Additionally, there is a risk of mosaic expression, where the desired genetic modification is not uniformly present in all cell types.^[^ ^24^ ^]^ In this sense, it has been shown that MHCII transgenic mice may lead to severe immunological disturbances including stressed B cells with altered numbers and with a stronger T helper type 2 (Th2) immune response.^[^ ^25^, ^26^, ^27^ ^]^

We therefore decided to make new mouse strains with a more physiologic expression of human MHCII molecules. We introduce a set of novel knock‐in mouse models expressing the extracellular domains of DRA and DRB1, together with human CD74 (human invariant chain, Ii), enabling a more physiologically relevant expression of human MHCII molecules. For the present work, we used mice expressing the *04:01 and *04:02 alleles, believed to be disease‐associated versus protective for RA, respectively, either by forming unique peptide binding pockets,^[^ ^4^ ^]^ affecting the loading of the CLIP peptide of the invariant chain,^[^ ^28^ ^]^ or as source of peptides binding to DQ molecules.^[^ ^29^, ^30^ ^]^

These new models hold great potential for providing human MHCII models with better physiologic expression and function needed as a tool for studies of MHCII‐associated diseases.

## 2 Results

### 2.1 Physiologic Expression of MHCII/Invariant Chain With Human Ectodomains on Thymic Epithelial Cells

**Figure 1:**
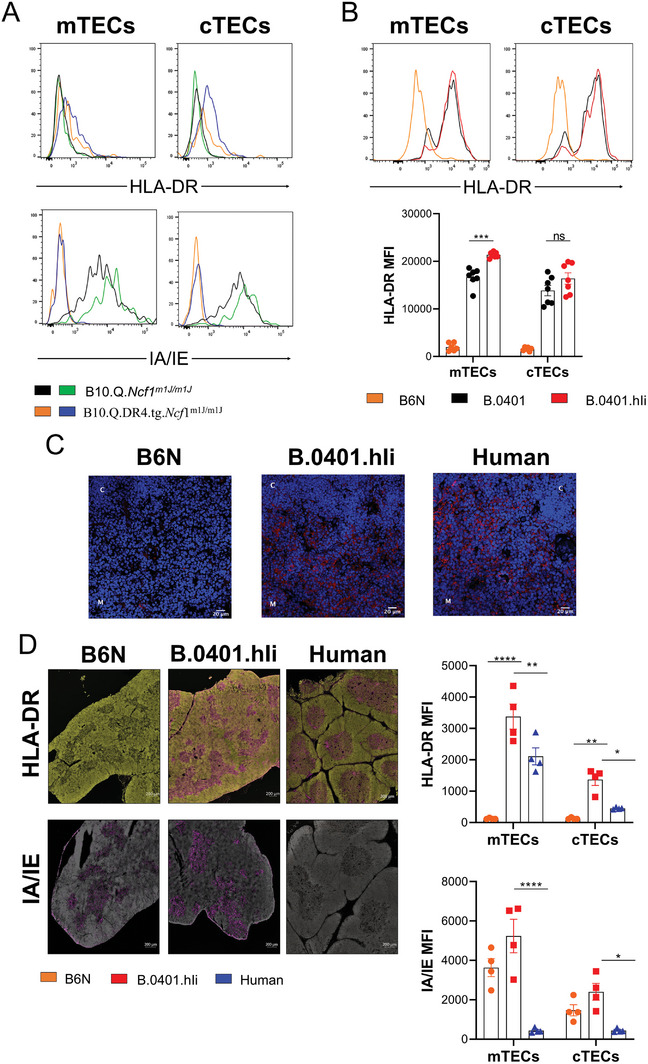
MHCII expression on thymus epithelial cells. A) Representative flow cytometry analysis depicting HLA‐DR and IA/IE expression on TECs in transgenic mice B10.Q.DR4fug.*Ncf1^m1J/m1J^* (*n =* 2) or B10.Q.*Ncf1^m1J/m1J^* mice (*n =* 2). B) Representative flow cytometry analysis depicting HLA‐DR expression on TECs in B.0401 (*n =* 7) and B.0401.hIi (*n =* 7) humanized mice and B6N mice (*n =* 6). C) Representative immunofluorescence images of HLA‐DR in thymic sections from B6N (*n =* 3), B.0401.hIi (*n =* 4) mice and human donors (*n =* 4). C = cortex, M = medulla. Red = HLA‐DR and Blue = Hoechst (nuclei). Scale bars equal 20 µm. D) Representative immunofluorescence images and the quantification of the signal intensity of HLA‐DR and IA/IE in thymic sections from B6N (*n =* 3), B.0401.hIi (*n =* 4) mice and human donors (*n =* 4). Scale bars equal 200 µm. Results are expressed as mean ± SEM. Statistics in (B and D) experiments represented were using a two‐way ANOVA with Sidak's multiple comparisons test.

The presence of MHCII molecules on thymic epithelial cells (TECs) is crucial to selecting functionally competent T lymphocytes and self‐tolerance.^[^ ^31^, ^32^ ^]^ Therefore, we investigated if these molecules are expressed on the TECs of our mouse strains. Interestingly, we found that the transgenic strain B10.Q.DR4.tg.*Ncf*1^m1J/m1J^ did not express HLA‐DR molecules properly on the TECs (**Figure** **1**A). In contrast, the B.0401 knock‐in humanized mice exhibited marked expression of human MHCII, and together with human Ii (B.0401.hIi) an even higher MHCII expression was observed (Figure 1B). This was expected given the vital role of the Ii in the intracellular formation and transport of the MHC class II molecules.^[^ ^33^, ^34^ ^]^

Analysis of human versus mouse thymi from B.0401.hIi mice show that HLA‐DR is expressed in both species (Figure 1C). However, the mean fluorescence intensity (MFI) of HLA‐DR was higher in B.0401.Ii mouse than in humans, which is likely related to general mouse versus human differences (Figure 1D). The levels of expression of both the mouse A^b^ (IA/IE) and chimeric DRB/DRA (HLA‐DR) in B.0401.Ii mice were the same as the level of expression of A^b^ (IA/IE) in B6N mice, indicating that the human MHCII was physiologically expressed (Figure 1D).

### 2.2 Human Invariant Chain (Ii) Improves Antigen Presentation

**Figure 2:**
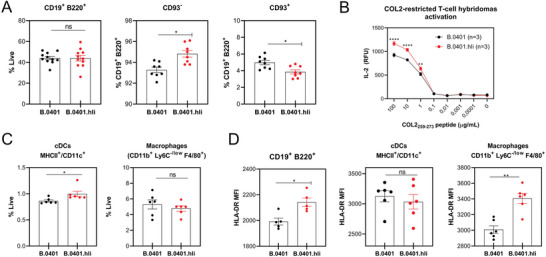
Higher numbers of matured B cells and enhanced antigen presentation in B.0401.hIi mice. A) Frequencies of total (CD19^+^ B220^+^), matured (CD93^−^), and immature (CD93^+^) B cells in naïve spleens from B.0401 and B.0401.hIi mice ((*n =* 11) for total B cells and (*n =* 8) for matured and immature B cells). B) Antigen presentation was assessed by measuring the IL‐2 titers from the supernatant by ELISA after co‐culture of native‐COL2‐specific T hybridoma (3H8) together with splenocytes from either B.0401 (*n =* 3) or B.0401.hIi (*n =* 3) mice, with or without COL2_259‐273_ peptide at different concentrations. Results are expressed as the mean ± SEM. C) Frequencies of conventional dendritic cells (cDCs [MHCII^+^ CD11c^+^]) and macrophages (CD11b^+^, Ly6C^−/low^, F4/80^+^) in naïve spleens from B.0401 (*n =* 6) and B.0401.hIi (*n =* 6) mice. D) Expression of HLA‐DR on B cells (CD19^+^ B220^+^); macrophages (CD11b^+^, Ly6C^−/low^, F4/80^+^) and cDCs (MHCII^+^ CD11c^+^) as determined by surface staining of spleen cells from naïve B.0401 and B.0401.hIi mice. ((*n =* 5) for B cells and (*n =* 6) for macrophages and cDCs). Expression was quantified as [MFI]. Results are expressed as mean ± SEM. Statistics in (A, C, and D) experiments represented were determined with a two‐tailed Mann‐Whitney test, and in (B) using a two‐ ANOVA with Sidak's multiple comparisons test.

The Ii chain plays an important role in the development and function of antigen‐presenting cells, including B cells.^[^ ^35^ ^]^ Therefore, we investigated whether there were differences in the total cell numbers of mature B cells derived from B.0401.hIi mice compared to B.0401 mice. The B.0401.hIi mice exhibited a higher number of mature B cells in the spleen than the B.0401 mice (**Figure** **2**A), but we did not observe any significant differences in the bone marrow between the two strains (Figure S1A, Supporting Information).

The binding of peptides and stability of MHCII is facilitated and regulated by the Ii,^[^ ^36^, ^37^ ^]^ and both the length and sequence of the human and mouse Ii differ. Therefore, the next step was to investigate differences in antigen presentation between B.0401 and B.0401.hIi mice. Our findings revealed that B.0401.hli splenocytes were more potent in activating native COL2‐restricted T‐cell hybridomas, as evidenced by increased IL‐2 secretion (Figure 2B). Notably, we observed no differences in T cell phenotype between the two strains in lymph nodes, spleen, or thymus (Figure S1B, Supporting Information), suggesting that the observed effect was not due to differences in the T cell subsets. Next, we investigated whether the enhanced antigen presentation in B.0401.hIi mice were due to an increase in the number of APCs or an elevation of HLA‐DR expression on the APCs. The B.0401.hIi mice exhibited a higher number of mature B cells (Figure 2A) and classical dendritic cells (cDCs) (Figure 2C) as well as an increase in the HLA‐DR expression on B cells and macrophages (Figure 2D).

### 2.3 Human Invariant Chain Enhances T Helper Type 1‐Mediated Inflammation and the Development of Arthritis

**Figure 3:**
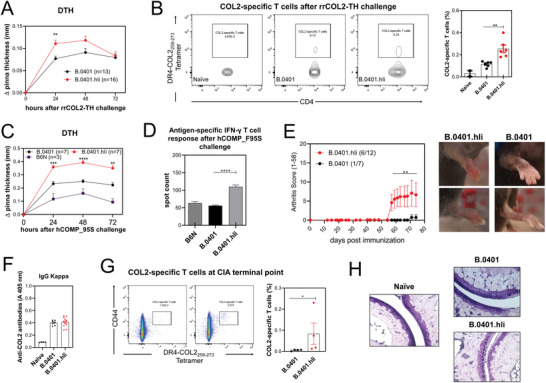
Human invariant chain enhances Th1‐mediated inflammation and the development of arthritis. A) Ear swelling of B.0401 (*n =* 13) and B.0401.hIi (*n =* 16) mice at indicated time points after rrCOL2‐TH challenge in the DTH model. Swelling was quantified as change (∆) in pinna thickness and presented as mean ± SEM. Data is representative of three experiments. B) Representative flow cytometry plots depicting the gating strategy in live/CD4^+^ cells and the frequency of COL2_259‐273_ T cells from lymph nodes and spleens of naïve (*n =* 2), B.0401 (*n =* 6) and B.0401.hIi (*n =* 6) mice 72 h after the rrCOL2‐TH challenge C) Ear swelling of B6N (*n =* 3), B.0401 (*n =* 7) and B.0401.hIi (*n =* 7) mice at indicated time points after hCOMP_F95S challenge. Swelling shown as mean ± SEM. D) Measurement of antigen‐specific IFNγ T cell response against the hCOMP_F95S protein, measured by ELISpot in inguinal lymph nodes 72 h after the hCOMP_F95S challenge. E) Left. CIA was induced using bovine collagen type II (bCOL2) in B.0401 (*n =* 7) and B.0401.hIi (*n =* 12) mice and inflammation in the paws was monitored over 73 days. Arthritis incidence is indicated in parenthesis. Data from two pooled experiments. Right. Pictures showing the arthritis phenotype of B.0401 and B.0401.hIi mice. F) Anti‐COL2 antibody titers of total IgG (anti‐kappa) from serum from naïve (*n =* 4) B.0401 (*n =* 7) and B.0401.hIi (*n =* 12) at arthritis endpoint measured by ELISA. Each dot represents an individual mouse. G) Representative flow cytometry plots and the frequency of COL2_259‐273_ T cells among the CD4^+^CD44^+^ cells from spleens from B.0401 (*n =* 4) and B.0401.hIi (*n =* 4) mice at arthritis endpoint. H) Representative histological samples of ankle joints taken at CIA endpoint. Sections were stained with toluidine blue (X20) Scale bars equal to 100 and 50 µm. Results are expressed as mean ± SEM. Statistics in (A, B, C, D, and F) experiments represented were determined using two‐ ANOVA with Sidak's multiple comparisons test, and in E and G were determined with a two‐tailed Mann‐Whitney test.

As mice express not only human MHCII but also the murine A^b^, it is important to study the functional effect mediated by these MHCII molecules separately. Although the COL2_259‐273_ peptide binds only the human MHCII, and not the A^b^ molecule, pepsin contamination in COL2 preparation has been shown to cause arthritis in B6N mice,^[^ ^38^ ^]^ an effect mediated by peptides from pepsin preferentially binding to A^b^. The delayed‐type hypersensitivity (DTH) model, a well‐established method that induces inflammation via IFNγ‐producing type 1 T helper (Th1) cells^[^ ^39^ ^]^ was used. To exclude the irrelevant response, we immunized B6N, B.0401, and B.0401.hIi mice with pepsin‐free recombinant rat collagen (rrCOL2‐TH). The absence of pepsin was verified by ELISpot (Figure S2A, Supporting Information). We also used recombinant human COMP containing a mutation at position 95 (hCOMP_F95S), blocking its ability to induce an immune response in the B6N mice.^[^ ^40^ ^]^ On day 8 post‐immunization, the mice were challenged by injection of the same antigen in the ear and subsequently, ear swelling was assessed at various time points. B.0401.hIi displayed increased ear pinna thickness 24‐ and 48 h post‐challenge when immunized with rrCOL2‐TH (**Figure** **3**A), along with higher numbers of COL2‐specific T cells at the termination point, as shown in Figure 3B. Similarly, when mice were immunized with hCOMP_F95S, ear swelling was higher in B.0401.hIi mice at 24, 48, and 72 h after challenge (Figure 3C) correlating with higher IFNγ secretion in these mice upon ex vivo stimulation (Figure 3D).

Next, we evaluated whether B.0401 and B.0401.hIi mice could develop CIA. As expected, B.0401.hIi mice exhibited increased arthritis incidence and severity compared to B.0401 mice (Figure 3E). No differences were observed in serum antibody levels against COL2 (Figure 3F). However, B.0401.hIi mice exhibited a higher percentage of COL2‐specific T cells at the terminal point of CIA in the spleen (Figure 3G). Histological analysis using toluidine blue staining, which enables the visualization of proteoglycans, showed a higher proteoglycan depletion in B.0401.hIi mice compared to naïve and B.0401 mice (Figure 3H), correlating with higher disease severity (Figure 3E).

Although B.0401.hIi mice did develop arthritis, it occurred at a late stage and with low incidence and severity. To enhance the susceptibility, a congenic fragment from the original *Mus musculus musculus* genome (denoted *Cia9i*), containing the FcR gene region, had been introduced into the genome of our previous strains;^[^ ^41^ ^]^ resulting in the creation of Parker (B6N.DRA.DRB1*0401.hIi.*Cia9i*) and Dunder (B6N.DRA.DRB1*0402.hIi.*Cia9i*) mice.

### 2.4 CIA Development and T Cell Response in Parker (DRB1*04:01) and Dunder (DRB1*04:02) Mice

**Figure 4:**
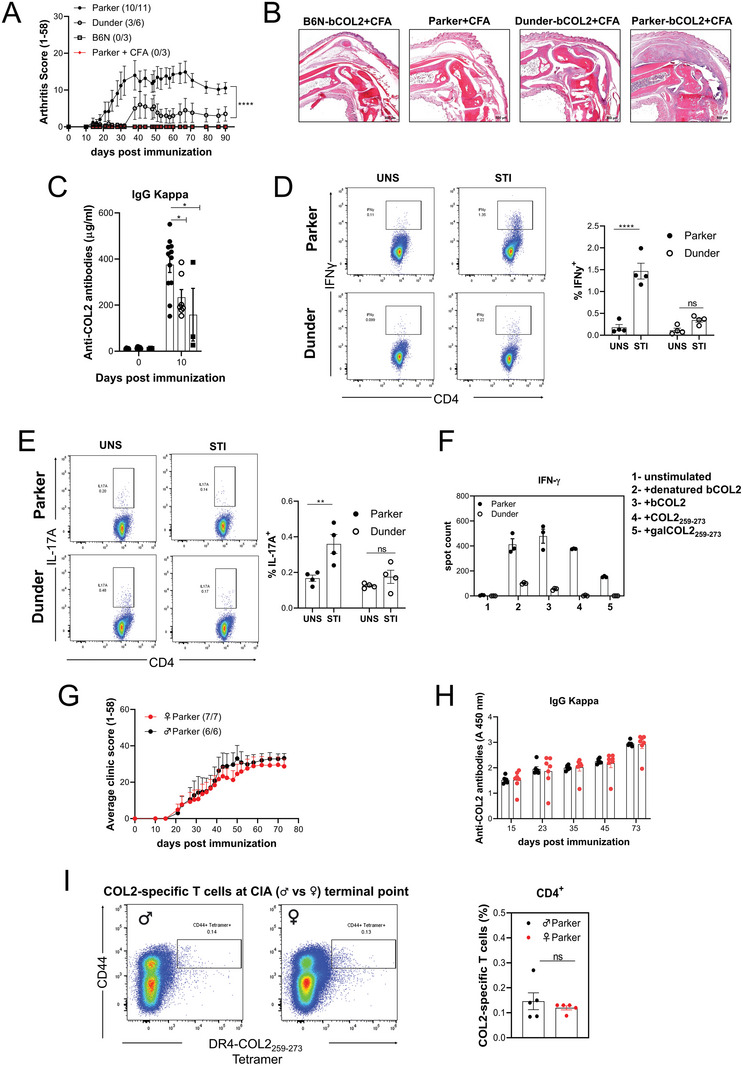
CIA development and T cell response in Parker (DRB1*04:01) and Dunder (DRB1*04:02) mice. A) Arthritis severity was evaluated in male B6N, Parker, and Dunder mice, following immunization with bCOL2 on day 0 and boosted on day 21. Inflammation on the paws was monitored over 90 days. Arthritis incidence and the total number of mice are indicated in parenthesis. B) Representative histological samples of ankle joints taken at CIA endpoint. Sections were stained with hematoxylin/eosin (H&E). Scale bars equal 800 µm. C) Anti‐COL2 antibody titer of total IgG (anti‐kappa) from serum at day 10 post‐immunization measured by ELISA. D) Left. Representative flow cytometry plots depict the gating strategy in live/CD4 cells for IFNγ‐ secreting T cells from Parker and Dunder mice after COL2 restimulation. Right. The percentage of IFNγ secreted by CD4+ T cells from Parker and Dunder mice after COL2 restimulation. E) Left. Representative flow cytometry plots depict the gating strategy in life/CD4 cells for IL‐17A‐secreting T cells from Parker and Dunder mice after COL2 restimulation. Right. The percentage of IL‐17A secreted by CD4+ T cells after COL2 restimulation from Parker and Dunder mice. F) Measurement of antigen‐specific IFNγ T cell response against native and denatured bCOL2, the native COL_253‐279_ peptide, and the gal‐COL2_253‐279_ measured by ELISpot in inguinal lymph nodes from Parker and Dunder mice 10 days post‐immunization. G) Arthritis severity was evaluated in male and female Parker mice, following immunization with bCOL2 on day 0 and boosted on day 35. Inflammation was monitored over 73 days. H) Anti‐COL2 antibody titer of total IgG (anti‐kappa) from serum at different time points of arthritis (G) measured by ELISA. I) Left. Representative flow cytometry plots depicting the gating strategy in live/CD4 cells for DR4‐COL2_259‐273_^+^/CD44^high^ T cells from male and female Parker mice at the terminal point of arthritis. Right. Frequency of DR4‐COL2_259‐273_^+^/CD44^high^ T cells from male and female Parker mice at the terminal point of arthritis. Each dot represents individual mice. Results are expressed as mean ± SEM. Statistics in (A, B, D, E, F, and H) experiments represented were determined using two‐ ANOVA with Sidak's multiple comparisons test, and in G and I were determined with a two‐tailed Mann‐Whitney test.

To determine whether the development of the immune response to COL2 was associated with the RA susceptibility of the MHCII allele DRB1*04:01 rather than the non‐associated DRB1*04:02, we immunized the Parker and Dunder mice with pepsin‐free bovine COL2, using B6N as a negative control, as well as Parker mice immunized with complete Freund's adjuvant (CFA) only Parker mice exhibited a high incidence and severity of arthritis, with approximately 90% of the mice affected (**Figure** **4**A), whereas only a few Dunder mice developed mild arthritis (Figure 4A), an effect confirmed with histology (Figure 4B). Likewise, the antibody response just before the development of arthritis (10 days after immunization) was higher in Parker, as compared with Dunder (Figure 4C). Importantly, the data presented in Figure 4A–C were replicated in an independent institution (Fraunhofer Institute) using the same strains of mice and antigen. In this experiment, the mice were kept in a conventional facility lacking SPF conditions, and boosting was performed 35 post‐primary immunizations instead of 21 days (Figure S3, Supporting Information).

Subsequently, we investigated the secretion of proinflammatory cytokines IFNγ and IL‐17A by T cells derived from bCOL2 immunized Parker and Dunder mice, following in vitro restimulation with COL2, using flow cytometry. Notably, our findings revealed a disparity in IFNγ secretion between Parker and Dunder mice upon COL2 restimulation (Figure 4D). Parker mice exhibited markedly higher levels of INFγ secretion, whereas this effect was absent in Dunder mice. Similar observations were made with regard to IL‐17A secretion, where only Parker mice demonstrated an increase in secretion following COL2 restimulation (Figure 4E).

To further elucidate the T cell‐specific response, we assessed the IFNγ secretion of Parker and Dunder mice 10 days after immunization with bCOL2, focusing on their responses to native and denatured bCOL2, the native COL_253‐279_ peptide, and the galactosylated COL2 peptide (gal‐COL2_253‐279_). Intriguingly, the data revealed a contrasting pattern in comparison to Parker mice. Dunder mice did not exhibit a discernible response to either native or gal‐COL2_253‐279_ peptides. However, they did display a weak response to the bCOL2 protein, indicating that 0402 might bind to other COL2 peptides, or possibly to pepsin contamination (Figure 4F). To address if pepsin could play a role, Dunder and Parker mice were immunized with bCOL2 and subsequently, the response of IFNγ‐secreting T cells upon pepsin stimulation was examined. Notably, Dunder mice exhibited a higher response compared to Parker mice following pepsin stimulation after bCOL2 immunization (Figure S2B, Supporting Information). This finding suggests that the heightened response of Dunder mice to COL2 might be mediated by pepsin rather than being specific to COL2 itself.

Despite the higher prevalence of RA in women, male mice are well known to be more susceptible to most RA models, including CIA.^[^ ^42^ ^]^ To examine the development of arthritis in both females and males, Parker mice were immunized with bCOL2. Remarkably, both genders exhibited comparable levels of arthritis incidence and clinical scores (Figure 4G). Furthermore, there were no discernible differences in the titers of COL2 autoantibodies between male and female Parker mice (Figure 4H). Additionally, the frequency of COL2‐specific T cells at the terminal stage of the CIA model did not vary significantly between male and female Parker mice (Figure 4I). Thus, compared with other arthritis mouse models, female humanized mice seem to be more susceptible to arthritis. This makes the humanized MHCII mice a valuable tool for dissecting gender‐specific differences in autoimmune diseases.

### 2.5 Parker.COL2^266E^ Mice have Strong T Cell Tolerance Reducing Arthritis Susceptibility, whereas Parker.COL2^264R266E^ Mice Develop Severe Arthritis

In the immunodominant COL2_259‐273_ peptide, the key TCR interaction site is a lysine at 264 positions (K264), which can be hydroxylated and glycosylated.^[^ ^43^ ^]^ Mouse COL2 differs from non‐self COL2 at position 266 by one aspartic acid (D266). The non‐self COL2 has glutamic acid (E266) binding 12x tighter to MHCII, explaining efficient presentation and a stronger post‐immunization response.^[^ ^22^, ^44^ ^]^ To investigate the mechanism of T cell tolerance operating in the CIA model, we have generated a mouse strain, Parker.COL2^266E^ (P.266E), with homozygous D266E mutations, leading to a complete replacement of aspartic acid (D) with glutamic acid (E) at position 266 in the immunodominant COL2_259–273_ peptide. In addition, we also made the Parker.COL2^264R266E^ (P.264R) strain that has both the K264R and the D266E mutation, leading to a change in the TCR recognition site by replacing lysine (K) with arginine (R) at position 264, which prevents T cell recognition of the COL2 peptide.^[^ ^22^ ^]^

**Figure 5:**
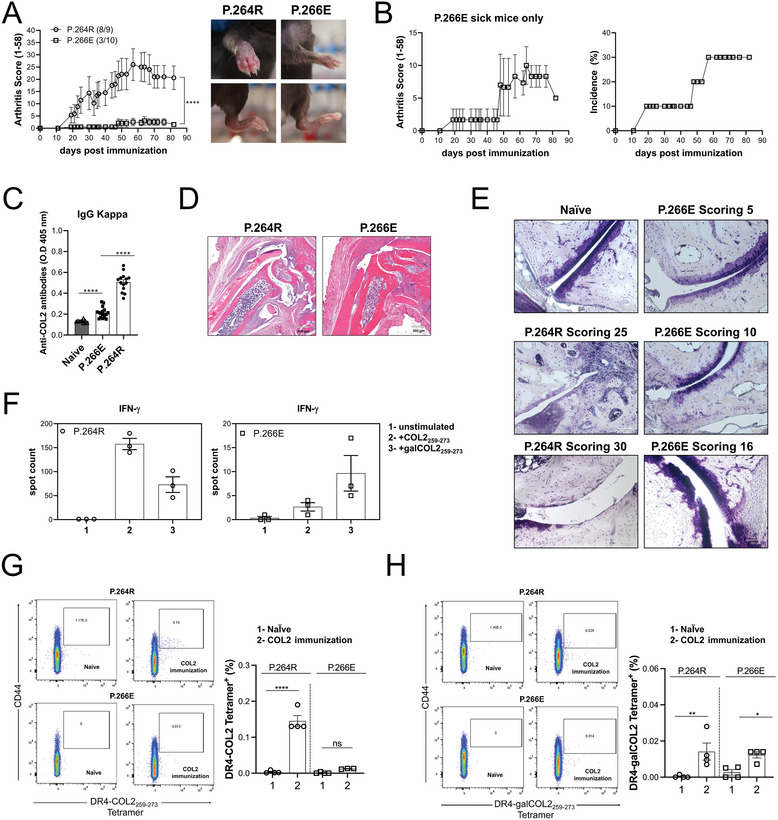
Parker.COL2^266E^ (P.266E) mice have strong T cell tolerance reducing arthritis susceptibility, whereas Parker.COL2^264R266E^ (P.264R) mice develop severe arthritis. A) Left. Arthritis severity was evaluated in male P.266E and P.264R mice, following immunization with bCOL2 on day 0 and boosted on day 35. Inflammation on the paws was monitored over 83 days. Arthritis incidence and total number of mice are indicated in parenthesis. Data from two pooled experiments. Right. Pictures showing the arthritis phenotype of P.266E and P.264R mice. B) Left. Arthritis severity in sick P.266 mice. Right. Arthritis incidence in P.266E mice. C) Anti‐COL2 antibody titer of total IgG (anti‐kappa) from serum at terminal point of arthritis measured by ELISA. D) Representative histological samples of ankle joints taken at CIA endpoint. Sections were stained with hematoxylin/eosin (H&E). Scale bars equal 500 µm. E) Representative histological samples of ankle joints taken at CIA endpoint. Sections were stained with toluidine blue. Scale bars equal 100 and 50 µm F) Left. Measurement of antigen‐specific IFNγ T cell response against native COL_253‐279_ peptide, and the gal‐COL2_253‐279_ measured by ELISpot in inguinal lymph nodesfrom bCOL2 P.264R mice. Right. Measurement of antigen‐specific IFNγ T cell response against native COL_253‐279_ peptide, and the gal‐COL2_253‐279_ measured by ELISpot in inguinal lymph noces from bCOL2 P.266E mice. G) Left. Representative flow cytometry plots depict the gating strategy in life/CD4 cells for DR4‐COL2_253‐279_^+^/CD44^high^ T cells from P.266E and P.264R mice after 10 days post‐COL2 immunization. Right. Frequency of DR4‐COL2_253‐279_^+^/CD44^high^ T cells from P.266E and P.264R mice after 10 days post‐COL2 immunization. H) Left. Representative flow cytometry plots depicting the gating strategy in live/CD4 cells DR4‐galCOL2_253‐279_^+^/CD44^high^ T cells from P.266E and P.264R mice after 10 days post‐COL2 immunization. Right. Frequency of DR4‐galCOL2_253‐279_^+^/CD44^high^ T cells from P.266E and P.264.R mice after 10 days post‐COL2 immunization. Each dot represents individual mice. Results are expressed as mean ± SEM. Statistics in C experiment represented were determined using two‐ ANOVA with Sidak's multiple comparisons test, and in (A, F, G, and H) were determined with a two‐tailed Mann‐Whitney test.

We evaluated the tolerance state, susceptibility to arthritis development, and ex vivo/in vivo T‐cell responses in P.266E and P.264R mice. The mice were immunized with bCOL2, and subsequent analysis revealed that P.264R mice developed severe arthritis, whereas P.266E mice exhibited clear, but milder, arthritis (**Figure** **5**A). To enhance the visualization of arthritis, we plotted the incidence and severity of P.266E mice during CIA, focusing solely on the affected individuals (Figure 5B). Moreover, to illustrate the dynamic nature of arthritis throughout the disease progression, we generated a heatmap depicting arthritis scores of each joint of an arthritic P.266E mouse (Figure S4, Supporting Information).

Measurement of COL2 autoantibody levels at the terminal stage demonstrated higher titers in P.264R mice compared to P.266E mice. However, P.266E mice still displayed higher levels of COL2 autoantibodies compared to naïve mice (Figure 5C). Histological examination corroborated arthritis development in both strains, including bone and cartilage destruction and active synovial inflammation, but with higher severity in the P.264R mice (Figure 5D,E).

We further investigated the T cell‐specific responses to the unmodified COL2_253‐279_ peptide and the galactosylated COL2_253‐279_ peptide using IFNγ ELISpot. T cells from the P.264R mice showed high reactivity against both native and galactosylated COL2 peptides (Figure 5F left). The T cell response elicited by the immunogen in question is not autoreactive, as the key endogenous T cell recognition epitope is not present in vivo. The P.266E mice showed a very low response, but which is autoreactive (Figure 5F right).

To confirm these results, we employed DR4‐COL2_259‐273_ and DR4‐galCOL2_259‐273_ tetramer staining and analyzed T cells from inguinal lymph nodes of naïve and bCOL2‐immunized P.266E and P.264R mice. As we predicted, the immunization of the P.264R mouse resulted in the expansion of T cells specific to both galactosylated and non‐modified 259–273 peptides, demonstrating their strong non‐self‐reactivity (Figure 5G). Interestingly, immunization of the P.266E mice, which triggers a self‐reactive T cell response, resulted in the development of similar levels of T cells to the gal 259–273 peptides as the P.264 mice, but with an undetectable response to the non‐modified peptide. We conclude that the galactosylated peptide escaped tolerance induction and is responsible for the development of arthritis (Figure 5H).

These results demonstrate that P.266E mice, expressing the immunodominant T‐cell epitope of rat/human COL2, exhibit T‐cell tolerance upon immunization with bCOL2 and display reduced susceptibility to arthritis compared to non‐tolerized P.264R mice.

## 3 Discussion

In this study, new mouse models incorporating human immune response genes were used to analyze the role of MHCII genes in autoimmune diseases like RA. In these models, human MHCII molecules were physiologically expressed, facilitating antigen processing, presentation, and induction of T‐cell tolerance. The development of CIA, a model for RA, showed a specific association with the DRB1*04:01 allele, emphasizing its importance in disease susceptibility, and confirming its suggested key importance for the HLA association of RA. Humanization of the target autoantigen, COL2, revealed activation of autoreactive COL2 specific and DRB1*04:01 restricted T cells which preferentially recognized the galactosylated COL2 peptide 259–271, rather than the corresponding non‐modified peptide. Thus, despite the physiological expression of human MHCII molecules in the thymus, a subset of the COL2 self‐reactive T cells evades thymic selection, which is potentially arthritogenic.

These findings show that the new humanized MHCII mouse models are useful to experimentally validate genetic polymorphisms suggested through human association studies, as well as investigating their functional implications. This holds significant indications for understanding the role of the MHC region polymorphisms in autoimmune diseases, given its gene‐dense nature, inherited as naturally selected haplotypes, and the presence of several pivotal genes.

While numerous genes within the MHC region may contribute to autoimmune processes, our current models allowed us to address a specific difference between the DRB1*04:01 and the *04:02 alleles of only one amino acid, which determines arthritis susceptibility. Specifically, the lysine residue at position 71 in DRB1 facilitates the binding of the COL2 peptide at position 266 with glutamic acid, whereas the presence of glutamic acid in the *04:02 allele precludes this binding.^[^ ^8^ ^]^ Moreover, we showed that the COL2 266E amino acid is crucial for robust binding, and a direct comparison with mice possessing D266 clearly demonstrates the profound impact of tolerance induction. We have earlier shown that the COL2 is expressed in the thymus but is not properly posttranslational modified leading to a thymus escape of autoreactive A^q^‐restricted T cells reactive with the galactosylated COL2.^[^ ^10^ ^]^ Clearly, our present investigation affirms that the same mechanisms operate also in the humanized DRB1*04:01‐expressing mice. Taken together, our data confirms the hypothesis that the difference between the *04:01 and *04:02 molecules is due to the peptide binding rather than peptide export for binding to other MHCII molecules. Our findings diverge from earlier studies using transgenic expression of *04:01 and *04:02 molecules, where *04:02 exhibited complete resistance due to a dominant suppression effect leading to a Th2‐profiled immune response. These results align with previous observations of toxic effects associated with transgenic expression.^[^ ^45^ ^]^

We have confirmed that transgenic expression of MHCII leads to artifacts,^[^ ^25^, ^26^ ^]^ caused by toxic expression in B cells and a lack of expression in thymic epithelial cells. These observations limit the usefulness of MHCII transgenic models and provide a plausible explanation for their lack of tolerance to non‐modified COL2 and heightened susceptibility to various autoimmune disease models, as well as challenges encountered in the induction of tolerance. Thus, this finding could explain why the MHCII transgenic lacks tolerance to non‐modified COL2 and is very susceptible to a variety of models for autoimmune diseases as well as for induction of tolerance.^[^ ^16^, ^46^, ^47^, ^48^ ^]^ Interestingly, in certain models, for example, diabetes, the toxicity could give the reversed effect and mediate disease protection however, this apparent protective effect could be an artifact finding as well.^[^ ^26^, ^27^ ^]^

Another important lesson derived from our study underscores the need to address specific interactions, as the expression and function of human MHCII depend on interactions with the mouse invariant chain. The mouse invariant chain exhibits not only a distinct sequence of the MHCII binding CLIP peptide but is also located at a differing length, resulting in disturbed interactions with MHCII.^[^ ^49^, ^50^ ^]^ The introduction of a human ectodomain of the invariant chain rectifies this artifact. It has become clear that interactions will be a main obstacle in transferring human genetic polymorphism into the mouse models, particularly considering the intricate nature of cross‐species protein interactions.

While new technologies in genetic modification have more recently facilitated the possibility of genetically modifying mouse strains, it is essential to emphasize that biological insights remain integral in guiding and controlling.^[^ ^24^, ^51^ ^]^

An observation, that was surprising to us, was that male and female mice were equally susceptible to arthritis. It was surprising since we have earlier shown that CIA is much easier to induce in male mice,^[^ ^42^ ^]^ a phenomenon which could be explained by the strong suppressive effects of estrogens,^[^ ^52^ ^]^ in similarity with human RA,^[^ ^53^, ^54^ ^]^ indicating that the female preponderance in RA^[^ ^55^ ^]^ is likely to be due to other mechanisms. Thus, the present humanized models are also useful for studies of gender‐dependent mechanisms. We have used the CIA model in the humanized models to demonstrate the importance of human MHCII single nucleotide polymorphism. However, RA is not CIA, as the disease starts with autoimmunity to citrullinated proteins whereas autoimmunity to triple helical COL2 appears around onset and only in a minor subset of patients. In addition, the CIA models do not involve the activation of B cells producing the typical ACPA response seen in RA.^[^ ^13^ ^]^ Thus, the nature and timing of presentation of self‐peptides by MHCII in humans developing RA is still not known but the present humanized models could be of key importance to understand these events more precisely. Furthermore, the utility of these humanized mice extends beyond dissecting disease mechanisms, positioning them as vital tools for advancing future therapies against RA. Specifically, these models are needed to elucidate the mechanisms underlying immunotherapy aiding in the translation into clinical applications.^[^ ^46^, ^56^ ^]^ Beyond their application in immunotherapy, these models could also emerge as critical assets for exploring innovative tissue engineering applications.^[^ ^57^, ^58^ ^]^

The new findings presented in this work became feasible through the utilization of humanized mouse strains with a physiologic expression of the human MHCII. We believe that these humanized models hold significant promise for unraveling a diverse spectrum of MHCII‐associated diseases and facilitating the development of therapeutic interventions.

## 4 Experimental Section

### Generation of the Genetically Modified Mice—Knock‐in of HLA‐DRA, HLA‐DRB1*04:01 and *04:02 alleles, and CD74

Knock‐ins were generated by gene targeting using homologous recombination in embryonic stem (ES) cells and were custom‐made by Ozgene (Pty Ltd, Perth, Australia). For the humanization of the α chain of MHCII (HLA‐DRA), the C57BL/6N (B6N) mouse MHCII H2‐*Ea‐pseudogene* (ps) promoter was repaired, and simultaneously the endogenous mouse Ea ectodomain gene sequence was replaced with human DRA ectodomain gene sequence (Figure S5, Supporting Information) The resulting humanized protein (Repaired/Human) is encoded by exons 2–3 of the human genomic DNA sequence, and exons 1 and 4 of the mouse gene.  The C57BL/6N derived *Ea*‐ps‐targeted ES cell clones were archived and used for subsequent HLA‐DRB1 humanizations by double targeting. 

For the humanization of the β chain of MHCII (DRB1*04:01 or DRB1*04:02), *Ea*‐ps‐targeted ES cells were targeted to humanize the *Eb*1‐41 locus with human exons 2–3 from the DRB1*04:01:01 or DRB1*04:02:01 gene and resulted in humanization of the ectodomains. The mice express both DRB1*04:01:01/*Eb*1‐41 (short name B.0401) or DRB1*04:02:01/*Eb*1‐41 (short name B.0402), and DRA/*Ea*‐ps human‐mouse chimeric proteins in which the signal peptides and the transmembrane and cytoplasmic domains were of mouse origin, to facilitate proper function and interactions in mouse cells.

For the humanization of the extracellular protein domains of the CD74 (invariant chain, (Ii)) the mouse exons 2–8 (≈4 Kb) were replaced with an orthologous human genomic fragment (exons and introns). Cytoplasmic part of the chimeric polypeptide is of mouse origin, to retain cytoplasmic functionality of the protein in mouse cells (Figure S5, Supporting Information).

### Generation of the Genetically Modified Mice—Mice with Humanized MHCII and Invariant Chain

Invariant chain (CD74) humanized mice were crossbred with humanized α (DRA) and β (DRB1*04:01 or *04:02) chain MHCII mice (to establish a strain to express all three humanized proteins (short name B.0401.hIi or B.0402.hIi) (Figure S5, Supporting Information). They expressed the chimeric proteins melding the human extracellular components with the mouse intracellular elements to facilitate not only a human‐like antigen presentation and physiological expression but also preserved functionality within the murine context.

In addition, a congenic fragment from the original *Mus musculus musculus* genome (denoted *Cia9i*), containing the FcR gene region, had been introduced into the B.0401.hIi and B.0402.hIi strains by crossing them with B6N.*Cia9i* mice ((Figure S5, Supporting Information) in order to increase the susceptibility to arthritis (short name Parker for B.0401.hIi.Cia9i and short name Dunder for B.0402.hIi.Cia9i).^[^ ^41^ ^]^

### Generation of the Genetically Modified Mice—Mice with Collagen type II (*Col2a1*) Mutations

The knock‐in Parker.COL2^D266E^ (P.266E) and Parker.COL2^K264R^ (P.264R) mouse lines (Figure S5, Supporting Information) were generated by introducing point mutations at exon 22 (p.D466E, c.1398 T > A) or (p.K464R, c.1391A>G; p.D466E, c.1398 T>A) of Collagen Type II Alpha 1 Chain (*COL2a1*) gene via CRISPR/Cas9 technology, respectively (Shanghai Biomodel Organism Science and Technology Development Co., Ltd, China). 

### Generation of the Genetically Modified Mice—Experimental Animals

All the humanized mouse strains had a C57BL/6N background and carried the MHC H‐2b haplotype. It meant that besides expressing the human MHCII molecules they also expressed the murine A^b^ molecule. The genetic modifications and the breeding strategy of the humanized mice are shown in (Figure S5, Supporting Information). All MHCII and COL2 humanized mouse strain founders were provided by Vacara (Vacara.se).

In addition to the B6N mice used as controls, two mouse strains on B10.Q (expressing A^q^) genetic background, B10.Q.*Ncf1* ^m1J/m1J^ and B10.Q.DR4.tg.*Ncf*1^m1J/m1J^ were also used.^[^ ^38^ ^]^ The strain B10.Q.*Ncf*1^m1J/m1J^ has a mutation in the *Ncf1* gene resulting in a low oxidative burst and increased CIA susceptibility^[^ ^59^ ^]^ and it was crossed with the strain B10.Q.Tg(Cd3d‐CD4,Ea‐DRA*0101,Eb‐DRB1*0401)#Lfug(short name B10.Q.DR4.tg, a, transgenic mice that co‐express DR4 and the human CD4 receptor).^[^ ^60^ ^]^

In our experiments, 8–10 weeks old female or male mice were used for phenotype and DTH experiments, meanwhile 12–14 weeks old female or male mice were used for the CIA model. All the experiments were performed with age‐ and sex‐matched mice and in a blinded fashion.

Mice were kept under specific pathogen‐free (SPF) conditions in the animal house of the Section for Medical Inflammation Research, Karolinska Institute, Stockholm, Sweden. Animals were housed in individually ventilated cages containing wood shavings in a climate‐controlled environment with a 14‐h light‐dark cycle, fed with standard chow and water ad libitum. All mice were healthy and basic physiological parameters were not affected. The sample size for each experiment was determined in accordance with the principles of the 3R (reduce, refine, replace) framework, ensuring the minimum required for statistical power while prioritizing ethical considerations. Experimental procedures were approved by the ethical committees in Stockholm, Sweden. Ethical permit numbers: 12923/18 and N134/13 (genotyping and serotyping), N35/16, and 2660/2021 (DTH, CIA).

### Expression and Purification of Recombinant Rat COL2 Triple Helical Region

The recombinant rat COL2 triple helical region designated as rrCOL2‐TH, was designed according to Figure S6, Supporting Information. The rrCOL2‐TH consists of the native leader sequence for extracellular expression, the his‐tag for purification, the thrombin cleavage site for removal of his‐tag if required, the mouse IgG2a hinge region, the collagen alpha‐1 (II) chain triple helical region, and the cys‐knot. Both the hinge region and the cys‐knot sequences were used to facilitate the formation of a triple helical structure. The gene was synthesized at ThermoFisher Scientific in their standard cloning vector with *KpnI* and *XhoI* restriction sites at 5’ and 3’ ends. The synthesized genes were restriction enzymes digested using the FastDigest enzymes (ThermoFisher Scientific). The digested DNA fragments were cloned into the mammalian expression vector pcDNA3.4 (ThermoFisher Scientific) which was digested using the same restriction enzymes. After sequence verification, the plasmid was transfected into Expi393F cells (ThermoFisher Scientific) using the FectoPRO DNA transfection reagent (Polyplus transfection). The supernatants were harvested 6 days post‐transfection. The synthesized recombinant protein was captured using a 5 mL HisTrap Excel (Cytiva) affinity column. To the culture supernatant, Imidazole was added to the concentration of 10 mm and loaded to the column that had been equilibrated in the PBS containing 10^−2^ m Imidazole. After washing, the rrCOL2‐TH was eluted stepwise with PBS containing 4 × 10^−1^ m Imidazole. The protein was then concentrated and diafiltrated into PBS followed by sterile filtration. The protein concentration was determined by the BCA method (ThermoFisher Scientific) with native collagen as standard. The purified protein was analyzed by SDS‐PAGE under both reducing and non‐reducing conditions.

### Delayed‐Type Hypersensitivity (DTH)

Twelve‐week‐old mice were sensitized by intradermal injection of 10^−4^ g rrCOL2‐TH or hCOMP_F95S^[^ ^40^ ^]^ in 10^−4^ L of a 1:1 emulsion with CFA (BD, Difco) and 10^−2^ m acetic acid at the base of the tail. On day 8 after sensitization, the right ear was injected intradermally with 10^−5^ L rrCOL2‐TH or hCOMP_F95S in PBS (10^−3^ g mL^−1^) whilst the control left ear was injected with 10^−5^ L acetic acid in PBS. Ear swelling response was measured during 0, 24, 48, and 72 h after the challenge using a caliper. Change in ear thickness was calculated by subtracting the swelling of the PBS‐injected ear from the swelling of the rrCOL2‐TH or hCOMP_F95S‐injected ear normalized to day 0 thickness.^[^ ^61^ ^]^

### Collagen‐Induced Arthritis (CIA)

Bovine collagen type II (bCOL2) was prepared from the calf joint cartilage, by limited pepsin digestion, and further purified, as previously described.^[^ ^62^ ^]^ The mice were immunized with 10^−4^ g of bCOL2 in 10^−4^ L of a 1:1 emulsion with CFA and 10^−2^ m acetic acid (mixed by an emulsifier machine, BTB POWER‐Kit)^[^ ^63^ ^]^ injected intradermally at the base of the tail. Mice were challenged on day 21 or 35 with 5 × 10^−5^ g of bCOL2 in 5 × 10^−5^ L incomplete Freund's adjuvant (IFA) (BD, Difco) emulsion injected intradermally. The progression of arthritis was assessed by visually inspecting the paws, employing a macroscopic scoring system. In this system, visibly inflamed ankles or wrists were assigned five points each, while inflamed toes or fingers were accorded one point each.^[^ ^64^ ^]^

### Peptides

COL2 peptides containing 259–273 sequences of rat COL2 with a non‐modified lysine at position 264 (GIAGFKGEQGPKGEP) or with a Gal‐peptide: GIAGFK(Gal‐Hyl‐K)GEQGPKGEP) were synthesized, purified, and characterized, as previously described.^[^ ^43^, ^44^ ^]^

### Serum Antibody Determination

During the CIA disease course, blood samples were collected through the cheek bleeding technique on different days and serum samples were used for the analysis of the COL2‐specific antibody response by ELISA. NuncTM MaxiSorpTM ELISA plates were coated with bCOL2 10^−5^ g L^−1^ overnight at 4 °C. The next day serum samples were added (typically 1:1000 to 10 000 in PBS) and incubated for 2 h at room temperature. Antibody binding was detected either by using horseradish peroxidase (HRP)‐conjugated goat anti‐mouse total IgG (Jackson Immuno Research Laboratories) or bio‐anti kappa (made in‐house, clone 187.1). The 2,2‐azino‐di‐ [3‐ethylbenzthiazoline sulfonate] diammonium salt (ABTS tablets, Sigma #11204521001) or the 3,3′,5,5′‐Tetramethylbenzidine (TMB, Serum Diagnostica #S‐100‐TMB) was used as a substrate The absorbance value was measured at 405 or 450 nm respectively.^[^ ^65^ ^]^

### Preparation of Cell Suspension From Lymphoid Organs

Spleens or inguinal lymph nodes were mashed using a 10^−6^ L syringe plunger on a 4 × 10^−5^ m cell strainer (Falcon). The cell suspension was washed once in PBS and centrifuged at 350 × g for 5 min at room temperature. For spleen, red blood cells were lysed in 10^−6^ L of red blood cell lysis buffer for 1–2 min at room temperature (0.155 m NH_4_Cl, 1.2 × 10^−2^ m NaHCO_3_, 10^−4^ M EDTA). Thereafter, cells were washed in PBS and carefully transferred to a new 15 mL tube to get rid of debris. Cells were centrifuged and resuspended in 1–3 × 10^−6^ L Dulbecco's modified Eagle's medium (DMEM) (Gibco, Invitrogen) for counting in a Sysmex KX‐21N cell counter. 

### In Vitro Antigen Presentation Assay

Spleen cells (APCs) prepared from naïve B.0401 and B.0401.hIi mice were resuspended in complete DMEM containing DMEM+Glutamax (Gibco), 5% heat‐inactivated fetal bovine serum (FBS) (Gibco), 10^−5^ m Hepes (Sigma), 5 × 10^−5^ g L^−1^ streptomycin sulfate (Sigma), 6 × 10^−5^ g L^−1^ penicillin C (Sigma), 5 × 10^−5^ m β‐mercaptoethanol (Gibco). Splenocytes (5 × 10^5^) were co‐cultured with DR4‐restricted T cell hybridoma cells specific for non‐modified COL2_259‐273_ epitope (3H8) (5 × 10^4^), in a total volume of 2 × 10^−4^ L in U‐bottom 96‐well plates. The COL2_259‐273_ peptide was titrated, while the amount of T‐cell hybridoma and APCs were kept constant. As negative controls, T‐cell hybridomas incubated with APCs in only media were used. After 24 h cultured at 37 °C, supernatants were collected to assess antigen presentation by measuring the IL‐2 levels by ELISA. For that, flat 96‐well plates (Maxisorp, Nunc) were coated overnight at 4 °C with the IL‐2 capture antibody (5 × 10^−6^ g L^−1^ JES6‐IA12, in‐house produced) in PBS. Supernatants from cell culture were added and plates were incubated for 2 h at room temperature before washing (PBS‐Tween 0.05%) and adding the biotinylated detection antibody (2 × 10^−6^ g L^−1^ JES6‐5H4, in‐house produced) for 1 h at room temperature. Plates were washed and incubated for 30 min at room temperature with Eu‐labeled streptavidin (PerkinElmer; 1:1000) in buffer E (5 × 10^−5^ m Tris‐HCl, 0.9% [wt/vol] NaCl, 0.5% [wt/vol] BSA, 0.1% Tween 20, 20 × 10^−5^ M EDTA). After washing, DELFIA Enhancement Solution (PerkinElmer) was added, and fluorescence was read at 620 nm (Synergy 2, BioTek).^[^ ^66^ ^]^ 

### ELISpot

EMD Millipore Multiscreen 96‐well plates were coated overnight at 4 °C with the IFN‐γ capture antibody (5 × 10^−6^ g L^−1^ AN18, in‐house produced) in PBS. The coating solution was decanted, and 10^6^ lymph node cells were seeded per well. After culture, plates were washed (PBS‐Tween 0.01%), and biotinylated detection antibody (2.5 × 10^−6^ g L^−1^ R46A2, in‐house produced) was added in PBS for 1 h at room temperature. Plates were washed and Extravidin alkaline phosphatase (Sigma) was added at a 1:2500 dilution in PBS (30 min, room temperature). Plates were washed before adding Sigmafast BCIP/NBT (Sigma) substrate solution and incubating for 5 to 10 min. When spots became visible, plates were washed in water and counted using a CTL ImmunoSpot Analyzer. Depending on the experiment, cells were stimulated with: COL2_259‐273_ (10^−5^ g L^−1^, in‐house produced); gal‐COL2_259‐273_ (10^−5^ g L^−1^, in‐house produced); guanidine hydrochloride denatured hCOMP_F95S (10^−5^ g L^−1^); temperature denatured bCOL2 and rat COL2 (rCOL2); and Concanavalin A (ConA) 10^−6^ g L^−1^, Sigma).^[^ ^61^ ^]^

### Flow Cytometry and Tetramer Staining

Murine thymic epithelial cells from naïve B.0401, B.0401.hIi, B10.Q.*Ncf*1^m1J/m1J^, B10.Q.DR4.tg.*Ncf*1^m1J/m1J^ and B6N were isolated, identified, and purified, as previously described.^[^ ^67^ ^]^ Single‐cell suspensions from spleens and inguinal lymph nodes were obtained as previously described above. All centrifugation steps were carried out at 350 × g for 5 min at room temperature. To block Fc receptors, the cells were incubated in 25 × 10^−5^ L of FACS buffer (2% FCS in PBS) containing 10^−5^ g an anti‐CD16/CD32 mAb (2.4G2; in‐house produced) in 96‐well plates for 15 min at 4 °C. Samples were washed with 1.5 × 10^−4^ L of PBS and subsequently stained with the indicated antibodies diluted 1:200 or 1:300 in 5 × 10^−5^ L of FACS buffer at 4 °C for 20 min in the dark. Cells were washed once, fixed, and permeabilized for intracellular staining using BD Cytofix/Cytoperm (BD Biosciences) according to the manufacturer´s instructions and stained with antibodies at 1:200 final dilution in 5 × 10^−5^ L of permeabilization buffer (BD Biosciences) for 30 min at room temperature. 

Tetramers DR4‐COL2_259‐273_ and DR4‐galCOL2_259‐273_ were prepared by mixing the biotinylated MHCII‐peptide complexes with streptavidin‐R.phycoerythrin (Thermo Fisher Scientific, Eugene, OR, USA) at 4:1 molar ratio.^[^ ^68^ ^]^ First, splenocytes and inguinal lymph node cells were incubated with an anti‐CD16/CD32 mAb and 5×10^−5^ M Dasatinib (Santa Cruz Biotechnology, Santa Cruz, CA, USA) for 30 min at 37 °C. Then the cells were stained with either DR4‐COL2_259‐273_ and DR4‐galCOL2_259‐273_ tetramer (2 × 10^−5^ g L^−1^) in DMEM containing 5% FBS (Gibco, Invitrogen) in the presence of 5 × 10^−5^ m Dasatinib for 1 h at 37 °C. Cells were stained for surface markers and analyzed by Attune NxT (ThermoFisher). The gate strategies for TECs, B cells, dendritic cells, and macrophages are shown in Figure S7, Supporting Information).

### Antibodies

The following reagents were purchased from Biolegend: anti‐EpCam (G8.8), anti‐Ly51 (6C3), anti‐HLA‐DR (L243), anti‐CD45 (30‐F11), anti‐CD19 (6D5), anti‐CD93 (AA4.1), anti‐IgD (11‐26c.2a), anti‐Ly6C (HK1.4), anti‐CD3 (500A2). The following reagents were purchased from BD Biosciences: anti‐B220 (RA3‐6B2), anti‐IgM (11/41), anti‐I‐A/I‐E (2G9), anti‐CD11b (M1/70), anti‐CD11c (HL3), anti‐cKit (2B8), anti‐CD2 (RM2‐5), anti‐CD4 (RM45), anti‐CD25 (PC61), anti‐CD8 (53‐6.7). The anti‐F4/80 (BM8) and anti‐Foxp3 (FJK‐16s) were obtained from Invitrogen. The anti‐UEA was purchased from Vector Laboratories. Live/dead dyes for discrimination of dead cells were purchased from ThermoFisher and 3 different formats were used: green, near‐infrared, and violet. 

### Immunofluorescence

Fresh frozen OCT‐embedded 7 × 10^−6^ m thymic tissue sections on Superfrost Plus microscope slides (Thermo Fisher Scientific, Waltham, Massachusetts, US) were fixed in cold acetone, left to dry at room temperature and then stored at −20 °C until use. Before immunostaining, the sections were brought to room temperature and rehydrated in PBS. Unspecific epitopes were blocked using protein block (Agilent Dako, Santa Clara, US) with avidin/biotin blocking solution (Vector Laboratories, CA, US). The sections were lightly rinsed with PBS before incubation with biotin anti‐human HLA‐DR antibody (clone L243, ref: 307614, BioLegend, San Diego, USA) diluted 1:100 in PBS with 0.1% Saponin for 1 h at room temperature. The sections were washed in PBS before incubation with Streptavidin Alexa Flour 555 secondary antibody (diluted 1:500) and Hoechst 24580 (diluted 1:1000, Invitrogen, Massachusetts, US) for 30 min in the dark at room temperature. After the final washing steps with PBS, the sections were mounted using ProLong Gold antifade reagent (Invitrogen). Images were acquired using an Axioscan 7 slide scanning microscope equipped with a ZEISS Colibri 7 camera and a plan‐apochromat 20×/0.8 M27 objective. The same scan profile (Hoechst 24580/Alexa fluor 555) was used for all sections; light source: LED‐Module 385/567 nm, exposure time: 264 ms/7.95 ms, filter ex. wavelength: 430–470/570‐640. Image analysis was performed with ZEN software (blue edition) version 3.5. Regions of interest (medulla and cortex) were based on nuclei staining.

### Histological Analyses

Mice were euthanized at the end of CIA. Skinless hind paws were fixed for 15 days in 10% phosphate‐buffered formaldehyde. Fixed tissues were decalcified for 4–5 weeks in decalcification buffer (10% EDTA, 7.5% polyvinylpyrrolidone, 0.1 m Tris‐HCl, pH 6.95), followed by dehydration in 70% ethanol and paraffin embedding. Tissue sections were then stained with hematoxylin and eosin (H&E) or toluidine blue (Sigma). Sectioning and H&E staining were performed by Histocenter (Gothenburg, Sweden).

### Human Donors

Human thymus tissues were obtained from children undergoing open heart surgery at the Queen Silvia Children's Hospital in Gothenburg, Sweden. The studies of human thymic samples were approved by the Regional Ethical Review Board in Gothenburg (DNR‐217‐12).

### Statistics

Graphs were plotted as mean ± SEM and the statistical analysis was done with GraphPad Prism version 9.0 (GraphPad Software Inc., La Jolla, CA, USA) as specified in each figure legend. The significance level was set to 0.05 and the p‐values were indicated with asterisks (*p<0.05, **p<0.01, ***p<0.001, and ****p<0.0001).

## Conflict of Interest

R.H. is a founder and O.S. and Z.X. are employees of Vacara AB.

## Author Contributions

All authors were involved in drafting the article or revising it critically for important intellectual content, and all authors approved the final version to be published. L.R.‐C. and R.H. had full access to all the data in the study and took responsibility for the integrity of the data and the accuracy of the data analysis. L.R.‐C., V.U., O.S., O.E., H.B., and R.H. designed research; L.R.‐C., T.L., N.‐D.D., B.X., V.H., Z.X., C.S., A.O.‐C, and Z.S., performed research; L.R.‐C., T.L., N.‐D.D., V.H., and Z.S., analyzed data; and L.R.‐C. and R.H. wrote the paper.

## Supporting information

Supporting Information

## Data Availability

The data that support the findings of this study are available from the corresponding author upon reasonable request.
