# Supplementary material for: Human MHC Class II and Invariant Chain Knock‐in Mice Mimic Rheumatoid Arthritis with Allele Restriction in Immune Response and Arthritis Association
Source: Adv Sci (Weinh). 2024 Apr 11;11(23):2401513. doi: 10.1002/advs.202401513 (PMC11187888; doi:10.1002/advs.202401513)
Supplement: Supplementary file 1 — Supporting Information [file ADVS-11-2401513-s001.pdf]

ADVANCED SCIENCE
Open Access

Supporting Information

for *Adv. Sci.*, DOI 10.1002/advs.202401513

Human MHC Class II and Invariant Chain Knock-in Mice Mimic Rheumatoid Arthritis with Allele Restriction in Immune Response and Arthritis Association

Laura Romero-Castillo, Taotao Li, Nhu-Nguyen Do, Outi Sareila, Bingze Xu, Viktoria Hennings, Zhongwei Xu, Carolin Svensson, Ana Oliveira-Coelho, Zeynep Sener, Vilma Urbonaviciute, Olov Ekwall, Harald Burkhardt and Rikard Holmdahl**

Supporting information for

Human MHC class II and invariant chain knock-in mice mimic rheumatoid arthritis with allele restriction in immune response and arthritis association.

Laura Romero-Castillo[1]*, Taotao Li[1], Nhu-Nguyen Do[1,2], Outi Sareila[1,5], Bingze Xu[1], Viktoria Hennings[3], Zhongwei Xu[1], Carolin Svensson[1], Ana Oliveira-Coelho[1], Zeynep Sener[1], Vilma Urbonaviciute[1], Olov Ekwall[3], Harald Burkhardt[2,4], Rikard Holmdahl*[1,5]

Correspondence:
Laura Romero-Castillo
Laura.romero.castillo@ki.se

Rikard Holmdahl
Rikard.holmdahl@ki.se

This PDF file includes:

S1 to S7

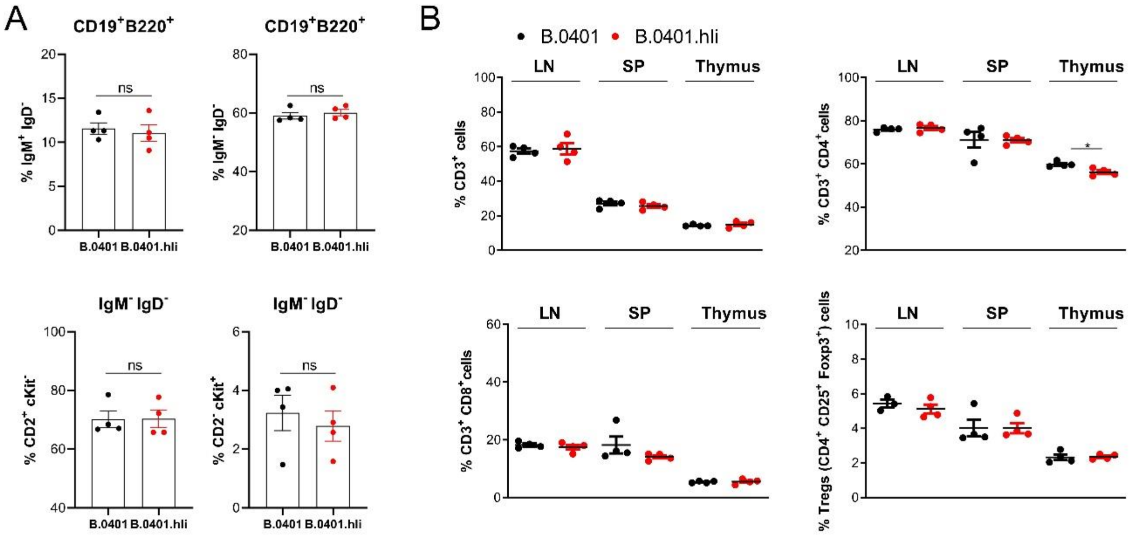

Figure S1– Unaffected immune cell populations in the presence of Ii in naive mice. (A) B cell development in the bone marrow with different B cell stages depicted (immature (IgM^+ IgD^-) pre and pro (IgM^- IgD^-), pre ($CD2^+$ $cKit^-$) and pro ($CD2^-$ $cKit^+$) B cells) from B.0401 (n = 4) and B.0401.hIi (n = 4) mice. (B) T cells in the lymph nodes, spleen, and thymus from B.0401 (n = 4) and B.0401.hIi (n = 4) mice. Each dot represents an individual mouse. Results are expressed as mean ± SEM. Statistics were determined using a Mann-Whitney test.

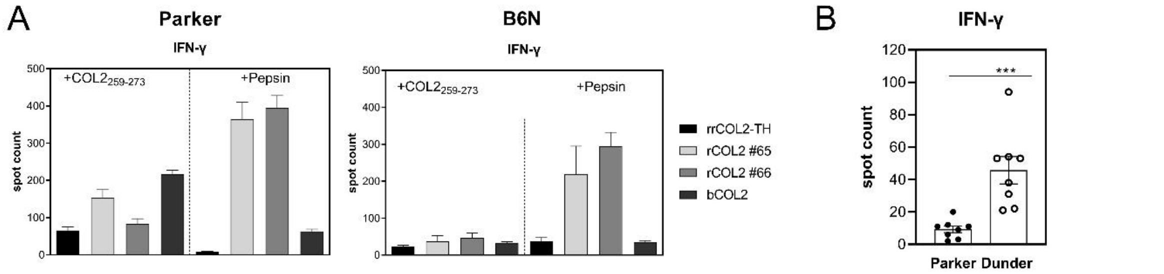

Figure S2– T cells response by COL2 and pepsin. (A) Parker and B6N mice were immunized with either rrCOL2-TH, different batches of rCOL2 (#65 and #66) or bCOL2, emulsified in CFA. On day 10 post-immunization, T cells from the iLNs were stimulated with either COL2$_{259-273}$ peptide or porcine pepsin and 24 h later the IFNγ-secreting T cells were detected by ELISpot. 2 mice per condition were used. **(B)** Dunder (n = 8) and Parker (n = 8) mice were immunized with bCOL2 emulsified in CFA. At day 10 post-immunization, T cells from the iLNs were stimulated with porcine pepsin and 24 h later the IFNγ-secreting T cells were detected by ELISpot. Each dot represents an individual mouse. Results are expressed as mean ± SEM. Statistics in B represented experiment were determined using a two-tailed Mann-Whitney test.

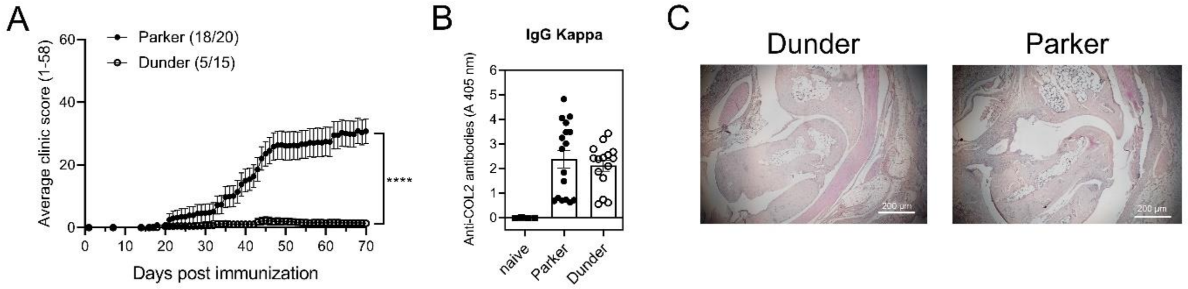

Figure S3– CIA development in Parker (0401) and Dunder (0402) mice in Fraunhofer Institute. (A) Arthritis severity was evaluated in male Parker (n = 20) and Dunder mice (n = 15), following immunization with bCOL2 on day 0 and boosted on day 35. Inflammation was monitored for over 70 days. Arthritis incidence is indicated in parenthesis. **(B)** Anti-COL2 antibody titer of total IgG (anti-kappa) from serum at terminal point measured by ELISA. Each dot represents individual mice. **(C)** Representative histological samples of ankle joints taken at CIA endpoint. Sections were stained with hematoxylin/eosin (H&E). Scale bars equal 20 µm. Results are expressed as mean ± SEM. Statistics in **(A)** experiment represented were determined with a two-tailed Mann-Whitney test.

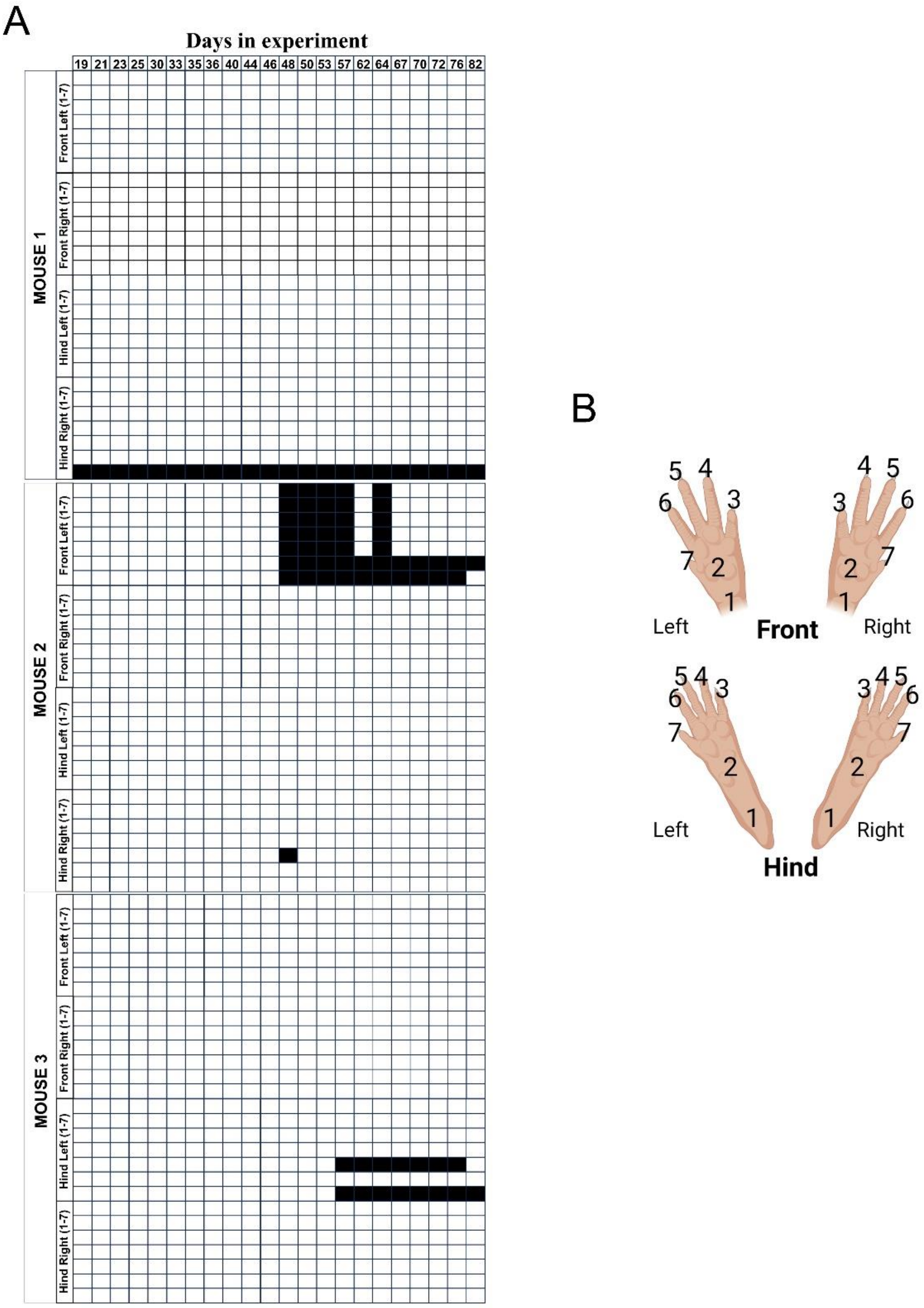

Figure S4– Affected joints during CIA model in the arthritic P.266E mice. To show the fluctuations in arthritis during the disease course, inflammation in each joint from a mouse with arthritis was plotted on a heatmap (A), in which each paw was numbered according to the scheme shown in B.

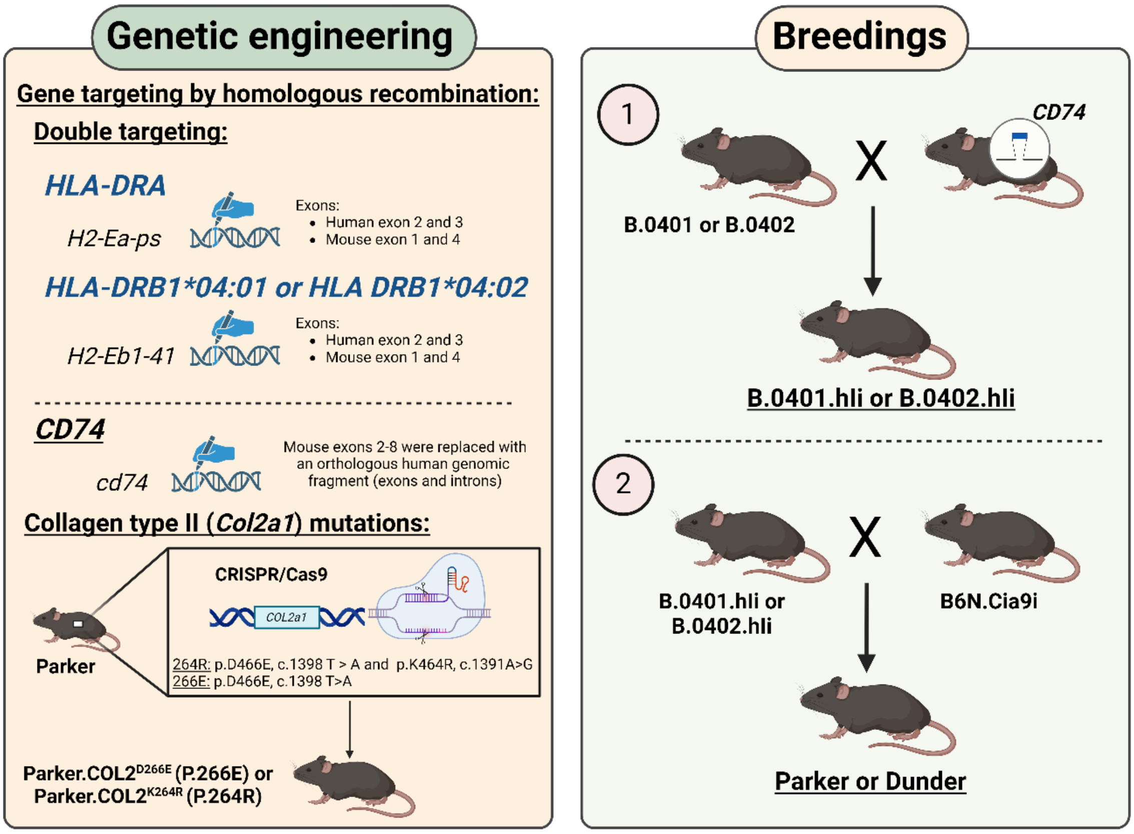

Figure S5- Genetic engineering and mouse breeding to obtain the humanized mice used in this study.

Sequence:

MIRLGAPQSLVLLTLLIATVLQCQGHHHHHHHHLVPRGSEPRGPTIKPCPPCKCPGPPGPPGPMGPMGPRGPPGPAGAPGPQGFQGNPGEPGEPGVSGPIGPRGPPGPAGKPGDD
GEAGKPGKAGERGLPGPQGARGFPGTPGLPGVKGHRGYPGLDGAKGEAGAPGVKGESGSPGENGSPGPMGPRGLPGERGRTGPAGAAGARGNDGQPGPAGPPGPVGPAGGP
GFLGAPGAKGEAGPTGARGPEGAQGSRGEPGNPGSPGPAGASGNPGTDGIPGAKGSAGAPGIAGAPGFPGPRGPPGPQGATGPLGPKGQTGEPGIAGFKGEQGPKGETGPAGP
QGAPGPAGEEGKRGARGEPGGAGPIGPPGERGAPGNRGFPGQDGLAGPKGAPGERGPSGLAGPKGANGDPGRPGEPGLPGARGLTGRPGDAGPQGKVGPSGAPGEDGRPGP
PGPQGARGQPGVMGFPGPKGANGEPGKAGEKGLAGAPGLRGLPGKDGETGAAGPPGPSGPAGERGEQGAPGPSGFQGLPGPPGPPGEGGKQGDQGIPGEAGAPGLVGPRGE
RGFPGERGSPGAQGLQGPRGLPGTPGTDGPKGAAGPDGPPGAQGPPGLQGMPGERGAAGIAGPKGDRGDVGEKGPEGAPGKDGGRGLTGPIGPPGPAGANGEKGEVGPPGPS
GSTGARGAPGERGETGPPGPAGFAGPPGADGQPGAKGDQGEAGQKGDAGAPGPQGPSGAPGPQGPTGVTGPKGARGAQGPPGATGFPGAAGRVGPPGSNGNPGPAGPPGPA
GKDGPKGARGDTGAPGRAGDPGLQGPAGAPGEKGEPGDDGPSGSDGPPGPQGLAGQRGIVGLPGQRGERGFPGLPGPSGEPGKQGAPGASGDRGPPGPVGPPGLTGPAGEPG
REGSPGADGPPGRDGAAGVKGDRGETGALGAPGAPGPPGSPGPAGPTGKQGDRGEAGAQGPMGPSGPAGARGIAGPQGPRGDKGEAGEPGERGLKGHRGFTGLQGLPGPPG
PSGDQGTSGPAGPSGPRGPPGPVGPSGKDGSNGIPGPIGPPGPRGRSGETGPAGPPGNPGPPGPPGPPGICDPSLC

Amino acids positions	Description
1 - 25	Native signal sequence
26 - 33	His-tag for purification
34 - 39	Thrombin cleavage site
40 - 55	Mouse IgG2a hinge region
56 - 1075	Collagen alpha-1 (II) chain triple helical region
1076 - 1083	Cys-knot

Figure S6- The recombinant rat COL2 triple helical region (rrCOL2-TH) protein design with description of the regions at amino acids positions.

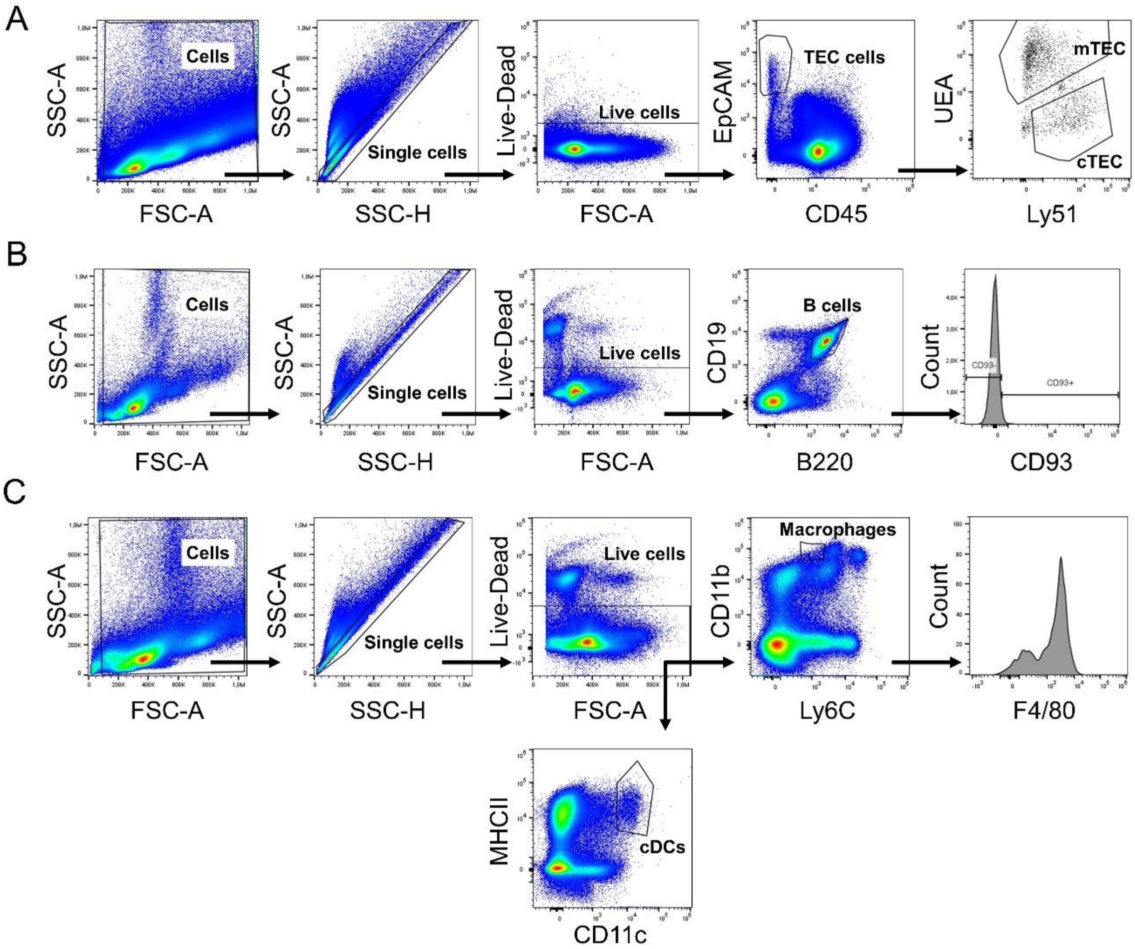

Figure S7- Gating strategy for FACS analyses of the main Figures 1 and 2.
